# Association between stress hyperglycemia ratio and acute total occlusion in patients with non-ST-segment elevation myocardial infarction

**DOI:** 10.3389/fendo.2026.1914746

**Published:** 2026-08-03

**Authors:** Shuai Wang, Jiatong Li, Shan Xie, Qicheng Yu, Haodong Jiang, Wenliang Zhai, Yanling Wang, Zhenxing Fan, Zhi Liu

**Affiliations:** 1 Department of Emergency, Xuanwu Hospital Capital Medical University, National Clinical Research Center for Geriatric Diseases, Beijing, China; 2 Department of Cardiology, Xuanwu Hospital Capital Medical University, Beijing, China; 3 Department of Geriatrics, Xuanwu Hospital Capital Medical University, Beijing, China

**Keywords:** acute total occlusion, coronary angiography, non-ST-segment elevation myocardial infarction, restricted cubic spline, stress hyperglycemia ratio

## Abstract

**Background:**

Acute total occlusion (ATO) of the culprit artery identifies a high-risk subgroup of patients with non-ST-segment elevation myocardial infarction (NSTEMI), but its early recognition remains challenging. The stress hyperglycemia ratio (SHR) reflects acute glycemic elevation relative to chronic glycemic status. This study investigated the association between admission SHR and ATO in patients with NSTEMI.

**Methods:**

This retrospective, single-center observational cohort study included 827 consecutive patients with NSTEMI who underwent coronary angiography between January 2017 and December 2023. SHR was calculated from fasting plasma glucose and glycated hemoglobin measured at admission. ATO was defined as Thrombolysis in Myocardial Infarction flow grade 0 or 1 in the culprit artery. Receiver operating characteristic analysis was used to assess the discriminatory performance of SHR, and multivariable logistic regression and restricted cubic spline analyses were performed to evaluate its independent and nonlinear associations with ATO.

**Results:**

In the overall cohort, SHR showed greater discriminatory performance for ATO (area under the curve, 0.743; 95% confidence interval [CI], 0.708–0.777) than fasting plasma glucose (FPG) (0.706; 95% CI, 0.670–0.742) or glycated hemoglobin (HbA1c) (0.571; 95% CI, 0.531–0.612). The optimal SHR cutoff was 0.903. After multivariable adjustment, SHR >0.903 remained independently associated with higher odds of ATO (adjusted odds ratio, 5.09; 95% CI, 3.66–7.06; P<0.001). Restricted cubic spline analyses further demonstrated significant overall and nonlinear associations between SHR and ATO, which remained robust after covariate adjustment and restriction to the 1st–99th percentile range (all P<0.001).

**Conclusion:**

In this retrospective cohort, elevated admission SHR was independently and nonlinearly associated with ATO. Although SHR may complement clinical assessment, further external validation is required before clinical implementation.

## 1 Introduction

NSTEMI is the most frequent form of acute myocardial infarction (AMI) and is associated with substantial morbidity and mortality (1, 2). Approximately one-third of patients with NSTEMI have acute occlusion of the culprit artery at coronary angiography (3). Such occlusion may occur without diagnostic ST-segment elevation, can be difficult to recognize from the presenting electrocardiogram, and is associated with adverse procedural and trial outcomes (4–6).

Metabolic disturbances, including hyperglycemia, are ubiquitous during acute cardiac events and exert deleterious effects on myocardial perfusion, platelet function, and coronary endothelial integrity (7, 8). Stress hyperglycemia—an acute rise in plasma glucose triggered by sympathoadrenal and counter-regulatory hormone activation—is distinct from pre-existing diabetes and chronic hyperglycemia. However, FPG alone cannot differentiate between acute stress-induced hyperglycemia and chronically elevated fasting glucose. The SHR provides a mathematically corrected index that captures the relative acuity of glycemic elevation beyond the chronic glycemic background (9).

Accumulating evidence suggests that elevated SHR independently predicts adverse outcomes in acute coronary syndromes, heart failure, and after percutaneous coronary intervention (PCI) (10–15). Nevertheless, the specific relationship between SHR and the occurrence of ATO in patients with NSTEMI—a distinct angiographic endpoint—has not been directly investigated. Given the clinical importance of recognizing ATO in NSTEMI, we hypothesized that SHR would be independently associated with ATO in this setting.

The present study aimed to: (1) characterize the glucometabolic profile of NSTEMI patients stratified by SHR; (2) identify the optimal SHR cut-off for predicting ATO using receiver operating characteristic (ROC) analysis; (3) assess the independent association of SHR for ATO by multivariable logistic regression; and (4) explore the dose-response relationship using restricted cubic spline analysis.

## 2 Materials and methods

### 2.1 Study design and population

The present study was a retrospective, single-center, observational cohort study conducted at Xuanwu Hospital, Capital Medical University, Beijing, China. We consecutively included eligible patients admitted with a diagnosis of NSTEMI between January 2017 and December 2023. NSTEMI diagnosis followed the Fourth Universal Definition of Myocardial Infarction (16), and patients were managed in accordance with contemporary acute coronary syndrome guidelines (1).

**Figure 1:**
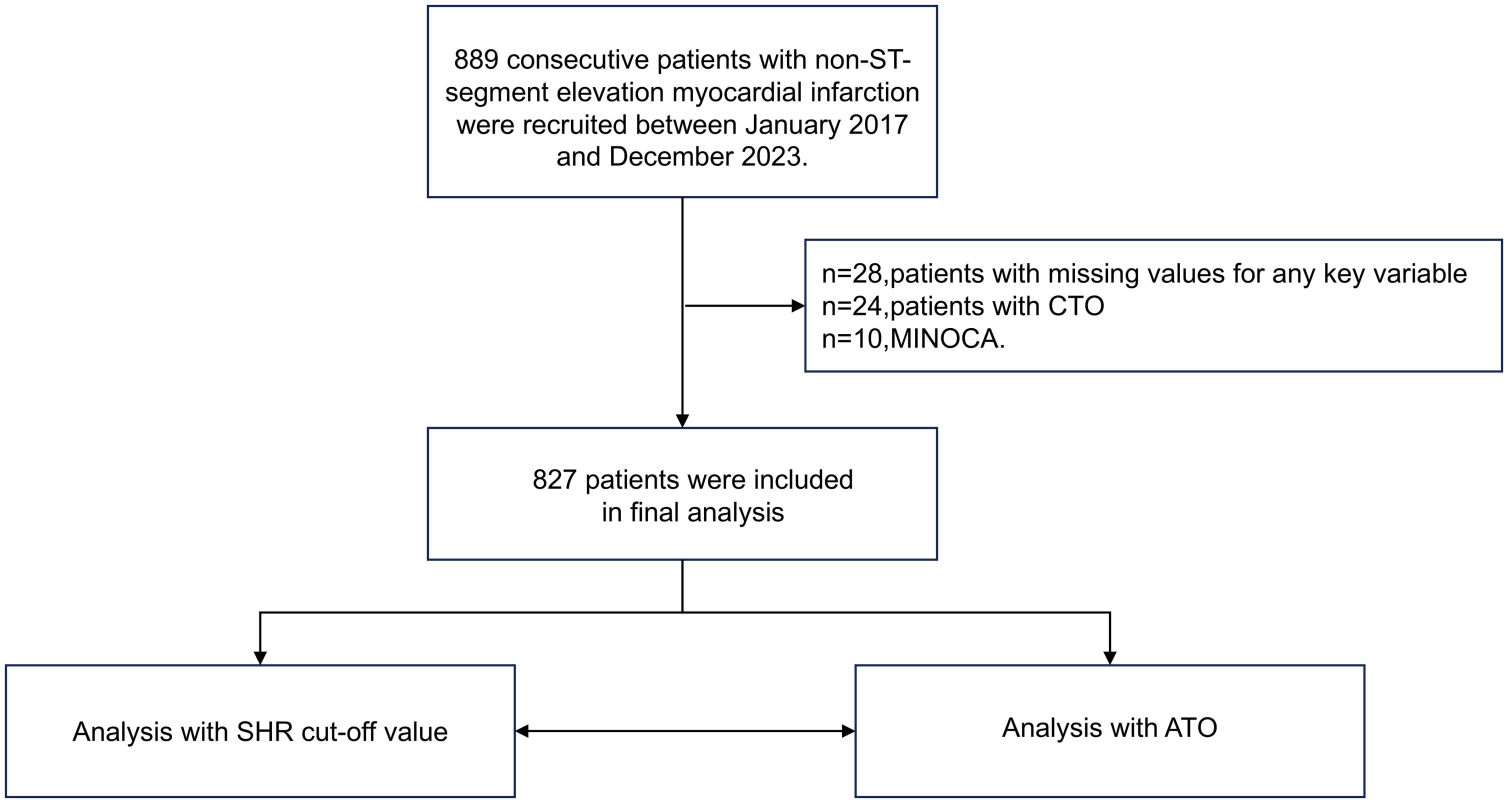
Flowchart of patient screening, exclusions, and final cohort selection.

Inclusion criteria: (1) age ≥18 years; (2) confirmed NSTEMI diagnosis; (3) undergone coronary angiography during the index hospitalization; (4) available FPG and HbA1c measurements at admission. Exclusion criteria: (1) missing values for any key variable. (2) chronic total occlusion (CTO). (3) non-obstructive myocardial infarction (MINOCA). Of 889 consecutive NSTEMI patients initially screened, 62 were excluded due to Exclusion criteria, yielding a final study cohort of 827 patients. A detailed flowchart is presented in **Figure 1**.

### 2.2 Definition of glucometabolic status

FPG and HbA1c were measured from the first venous blood sample obtained within 2 hours after emergency admission and before any documented in-hospital glucose-lowering treatment. The median blood-draw-to-angiography interval was 24 hours (interquartile range, 12–36 hours). All blood samples were obtained from the first venous draw at emergency admission, before any documented in-hospital glucose-lowering treatment. SHR was calculated using the validated formula: SHR = FPG (mmol/L)/[1.59 × HbA1c (%) − 2.59] (9). This formula estimates expected fasting glucose from HbA1c and expresses measured glucose relative to the chronic glycemic background. Among the 412 patients classified as having T2DM, 370 had a documented pre-existing diagnosis or were receiving glucose-lowering therapy, whereas 42 were newly identified during the index hospitalization. Newly identified T2DM was defined according to HbA1c ≥6.5% and/or confirmatory glucose testing rather than a single elevated admission glucose value. All cases were confirmed as type 2 diabetes mellitus, and use of diabetes drugs - was recorded. Patients were stratified by the optimal SHR cutoff identified by ROC analysis (SHR ≤0.903 vs. SHR >0.903).

### 2.3 Definition of ATO

The culprit artery was identified by the interventional cardiologist based on clinical, electrocardiographic, and angiographic criteria. Coronary flow was graded according to the Thrombolysis in Myocardial Infarction (TIMI) classification. ATO was operationally defined as pre-intervention TIMI flow grade 0 or 1 in the culprit artery at diagnostic angiography, consistent with previous NSTEMI studies. TIMI grade 1 indicates limited contrast penetration beyond the obstruction without complete opacification of the distal coronary bed and was therefore grouped with TIMI grade 0 as ATO (3, 4, 6). CTO was defined as TIMI grade 0 antegrade flow through the lesion with a documented or probable occlusion duration of at least 3 months. When previous coronary angiography or reliable clinical information regarding occlusion duration was unavailable, probable CTO was determined from the overall angiographic appearance, including a blunt proximal cap, mature or bridging collateral circulation, marked calcification, a long occluded segment, and the absence of visible thrombus or contrast staining at the proximal cap. No single angiographic feature, including collateral grade alone, was considered sufficient to establish chronicity.

All lesions with ATO in the presumed culprit artery and CTO were independently reviewed by two experienced interventional cardiologists who were blinded to the glucometabolic data. Disagreements were resolved by consensus.

### 2.4 Statistical analysis

Continuous variables were tested for normality using the Kolmogorov-Smirnov test. Normally distributed variables are expressed as mean ± standard deviation (SD) and compared using independent Student’s t-test. Non-normally distributed variables are expressed as median with interquartile range (IQR) and compared using the Mann-Whitney U test. Categorical variables are expressed as number with percentage and compared using the Pearson chi-square test or Fisher’s exact test as appropriate.

Receiver operating characteristic (ROC) curve analysis was used to determine the optimal cut-off values of FPG, HbA1c, and SHR for predicting ATO, with the optimal cut-off identified by maximizing the Youden index. Area under the curve (AUC) values were compared using the DeLong method.

Univariable and multivariable logistic regression was used to examine candidate factors associated with ATO. Because FPG and HbA1c are mathematical components of SHR and showed substantial collinearity with SHR, they were not entered simultaneously in the final SHR-based model. Results are reported as odds ratios (ORs) or adjusted ORs with 95% confidence intervals (CIs).

To explore the dose-response relationship between SHR and ATO, restricted cubic spline (RCS) analysis was conducted using three knots placed at the 10th, 50th, and 90th percentiles of the SHR distribution. All statistical analyses were performed using R software (version 4.3.1). A two-tailed p value <0.05 was considered statistically significant.

## 3 Results

### 3.1 Study population and glucometabolic status

**Figure 2:**
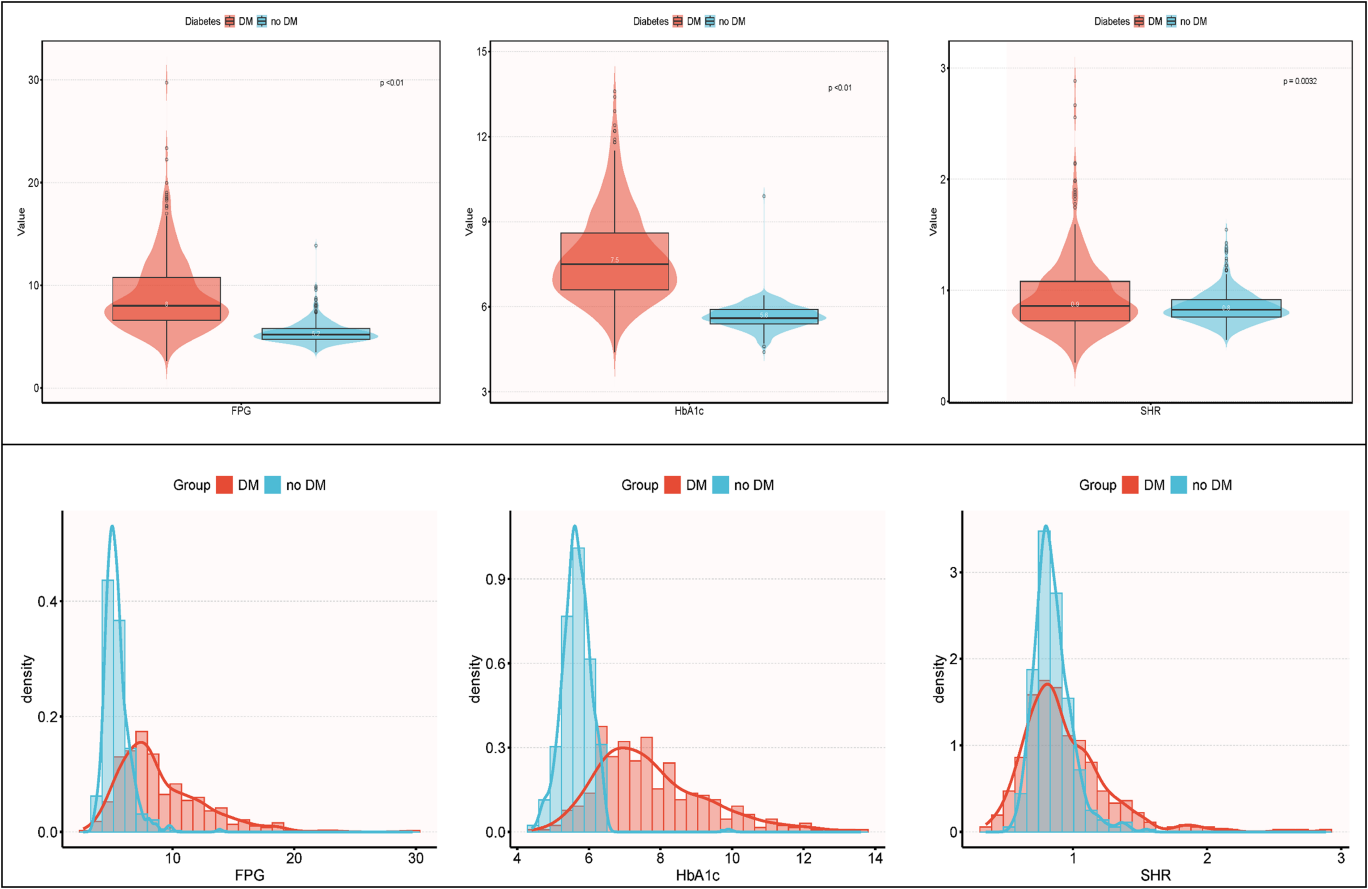
Admission glucometabolic profiles in patients with and without T2DM. Distributions of FPG, HbA1c, and SHR are shown by T2DM status.

A total of 827 NSTEMI patients were included in the final analysis. The distribution of admission glucometabolic parameters, stratified by the presence or absence of DM, is presented in **Figure 2**. Patients with DM demonstrated significantly higher levels of FPG, HbA1c, and SHR compared with their non-DM counterparts.

**Figure 3:**
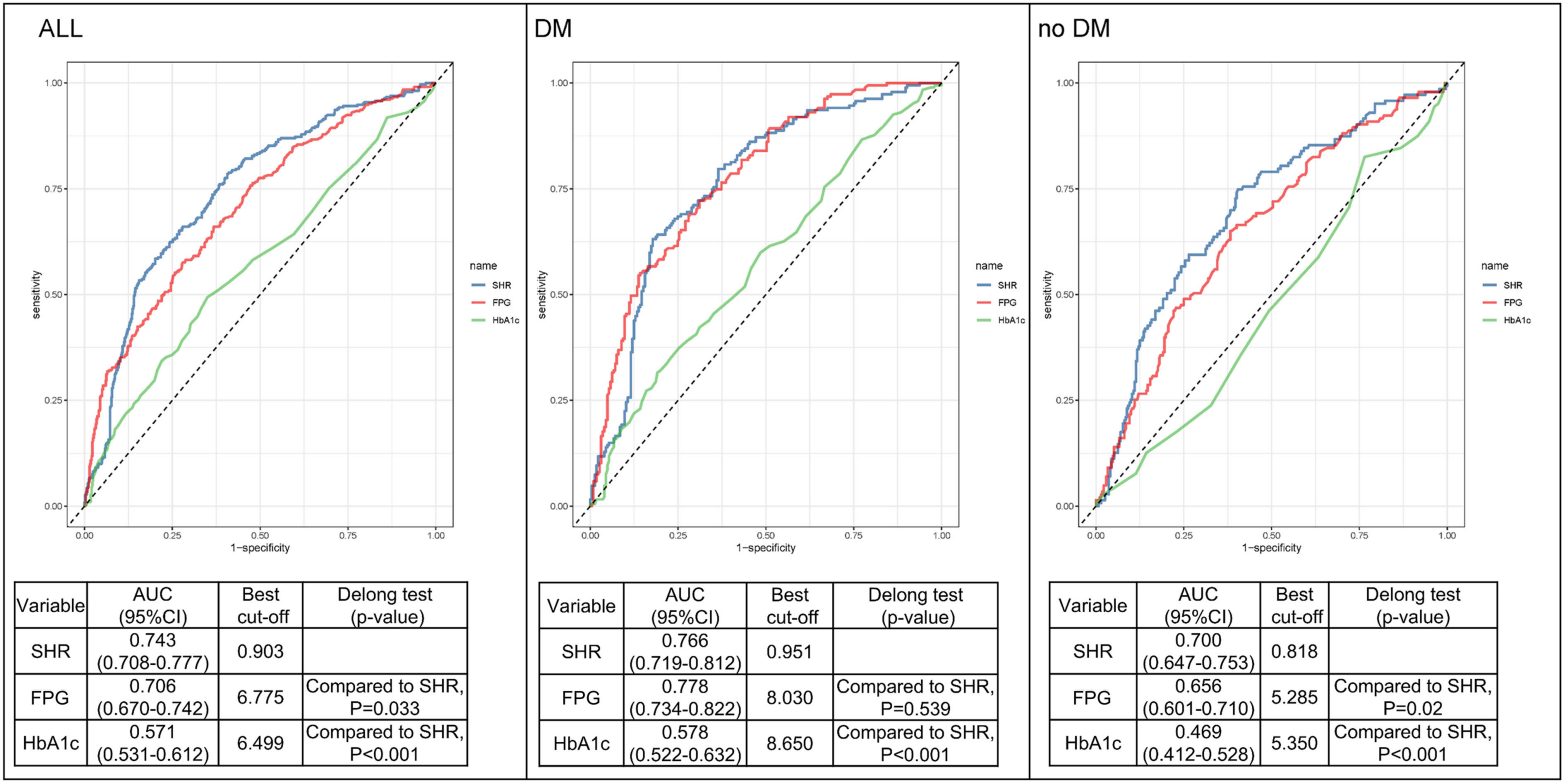
ROC curves for FPG, HbA1c, and SHR for discrimination of ATO in the overall cohort, patients with T2DM, and patients without T2DM. AUCs, 95% CIs, optimal cutoffs, and DeLong comparisons with SHR are shown.

For **Figure 3**, In the overall NSTEMI cohort, SHR demonstrated the highest AUC of 0.743 (95% CI: 0.708–0.777), compared with FPG (AUC 0.706, 95% CI: 0.670–0.742) and HbA1c (AUC 0.571, 95% CI: 0.531–0.612). In diabetic patients, SHR yielded an AUC of 0.766 (95% CI: 0.719–0.812), FPG showed an AUC of 0.778 (95% CI: 0.734–0.822), and HbA1c had an AUC of 0.578 (95% CI: 0.522–0.632). In non-diabetic patients, SHR achieved an AUC of 0.700 (95% CI: 0.647–0.753), FPG showed an AUC of 0.656 (95% CI: 0.601–0.710), and HbA1c had an AUC of 0.469 (95% CI: 0.412–0.528).

Baseline characteristics stratified by SHR are summarized in **Table 1**. A total of 827 patients with NSTEMI were included, of whom 534 (64.6%) had an SHR ≤0.903 and 293 (35.4%) had an SHR >0.903. The mean age of the overall population was 62.4 ± 10.8 years, and 650 patients (78.6%) were male. Compared with patients in the lower-SHR group, those with an SHR >0.903 had a significantly higher prevalence of diabetes mellitus and greater use of glucose-lowering medications. The incidence of acute total occlusion was markedly higher in the higher-SHR group than in the lower-SHR group, with significant differences also observed in the distribution of culprit vessels. Patients with an SHR >0.903 had higher levels of Hs-cTnI, creatine kinase-MB, and fasting plasma glucose, together with a lower left ventricular ejection fraction. In contrast, no significant between-group differences were found in age, sex, smoking or drinking history, hypertension, BMI, Killip class, time from symptom onset to admission, HbA1c, renal function, lipid profiles, white blood cell count, or myoglobin levels (all p>0.05).

**Table 1:** Baseline characteristics of the population stratified by SHR cut-off.

Variables	Total (n=827)	SHR<=0.903 (n=534)	SHR>0.903 (n=293)	P value
Age, years	62.4 ± 10.8	62.4 ± 10.3	62.3 ± 11.7	0.903
Sex Male, n(%)	650 (78.6)	422 (79)	228 (77.8)	0.685
Current/past smoking, n(%)	465 (56.2)	310 (58.1)	155 (52.9)	0.153
Current/past drinking, n(%)	530 (64.1)	346 (64.8)	184 (62.8)	0.567
Hypertension, n(%)	529 (64.0)	336 (62.9)	193 (65.9)	0.398
Diabetes, n(%)	412 (49.8)	231 (43.3)	181 (61.8)	< 0.001
*Confirmed T2DM among Diabetes*, *n(%)*	*370 (89.8)*	226 (61.1)	144 (38.9)	
*Newly diagnosed T2DM among Diabetes*, *n(%)*	42 (10.2)	5 (11.9)	37 (88.1)	
Diabetes drugs, n(%)	337 (40.7)	194 (36.3)	143 (48.8)	< 0.001
BMI, kg/m²	26.0 ± 3.4	25.9 ± 3.4	26.3 ± 3.5	0.063
Killip class≥2, n(%)	216 (26.1)	138 (26.5)	78 (28)	0.655
Time from onset to admission, h	24.0 (14.5, 48.0)	24.0 (14.2, 48.0)	24.0 (15.0, 48.0)	0.716
Culprit vessel with ATO, n(%)	330 (39.9)	137 (25.7)	193 (65.9)	< 0.001
*LAD among ATO*, *n(%)*	69 (20.9)	24 (17.5)	45 (23.3)	
*LCX among ATO*, *n(%)*	163 (49.4)	65 (47.4)	98 (50.8)	
*RCA among ATO*, *n(%)*	98 (29.7)	48 (35.0)	50 (25.9)	
Hs-cTnI, ng/L	0.4 (0.1, 1.6)	0.3 (0.1, 1.2)	0.5 (0.1, 2.2)	0.013
CK-MB, IU/L	8.4 (3.0, 33.0)	8.0 (2.9, 27.2)	10.6 (3.1, 40.9)	0.037
MYO, µg/L	40.0 (20.5, 97.1)	39.0 (20.4, 90.9)	41.0 (21.6, 115.0)	0.175
FPG, mmol/L	7.2 ± 3.2	5.9 ± 1.7	9.5 ± 3.9	< 0.001
HbA1c, %	6.7 ± 1.6	6.6 ± 1.5	6.8 ± 1.6	0.055
Creatinine, μmol/L	77.0 ± 40.8	76.1 ± 38.1	78.6 ± 45.4	0.402
Urea, mmol/L	5.7 ± 2.4	5.7 ± 2.4	5.8 ± 2.4	0.638
HDL-C, mmol/L	1.0 ± 0.3	1.0 ± 0.3	1.0 ± 0.3	0.549
LDL-C, mmol/L	2.6 ± 0.8	2.6 ± 0.9	2.6 ± 0.8	0.495
WBC, ×10^9^/L	8.4 ± 2.6	8.4 ± 2.5	8.4 ± 2.7	0.939
LVEF, %	59.4 ± 9.7	60.3 ± 9.0	57.7 ± 10.8	< 0.001

### 3.2 Logistic regression analysis for factors associated with ATO

Univariate logistic regression identified the following variables as significantly associated with ATO (**Table 2**). Current or past drinking, diabetes, use of glucose-lowering medications, BMI, hs-cTnI, CK-MB, myoglobin, FPG, HbA1c, HDL-C, LVEF, and SHR >0.903 were significantly associated with ATO (all P<0.05). Among these variables, SHR >0.903 showed the strongest association with higher odds of acute total occlusion (OR, 5.59; 95% CI, 4.10–7.62; P<0.001). Higher fasting plasma glucose (OR, 1.29; 95% CI, 1.22–1.36; P<0.001), HbA1c (OR, 1.20; 95% CI, 1.10–1.31; P<0.001), hs-cTnI (OR, 1.08; 95% CI, 1.03–1.14; P = 0.002) and BMI (OR, 1.05; 95% CI, 1.01–1.09; P = 0.027) were also associated with higher odds of acute total occlusion. Conversely, higher HDL-C (OR, 0.49; 95% CI, 0.28–0.86; P = 0.014) and LVEF (OR, 0.97; 95% CI, 0.96–0.99; P<0.001) were associated with lower odds of ATO.

**Table 2:** Univariate logistic regression analysis of risk factors associated with ATO.

Variable	OR (95% CI)	P value
Age	1 (0.98–1.01)	0.47
Sex (Male)	0.92 (0.66–1.3)	0.649
Current/past smoking	1.24 (0.94–1.64)	0.135
Current/past drinking	0.73 (0.55–0.97)	0.032
Hypertension	1.04 (0.78–1.39)	0.777
Diabetes	0.63 (0.48–0.84)	0.001
Diabetes drugs	1.35 (1.02–1.79)	0.036
BMI	1.05 (1.01–1.09)	0.027
Killip class ≥2	1.26 (0.92–1.73)	0.154
Time from onset to admission	1 (0.99–1)	0.487
Hs-cTnI	1.08 (1.03–1.14)	0.002
CK-MB	1 (1–1.01)	0.001
MYO	1 (1–1)	0.016
FPG	1.29 (1.22–1.36)	<0.001
HbA1c	1.2 (1.1–1.31)	<0.001
Creatinine	1 (1–1.01)	0.098
Urea	1 (0.94–1.06)	0.992
HDL-C	0.49 (0.28–0.86)	0.014
LDL-C	1.1 (0.94–1.3)	0.238
WBC	1.05 (1–1.11)	0.063
LVEF	0.97 (0.96–0.99)	<0.001
SHR > 0.903	5.59 (4.1–7.62)	<0.001

**Figure 4:**
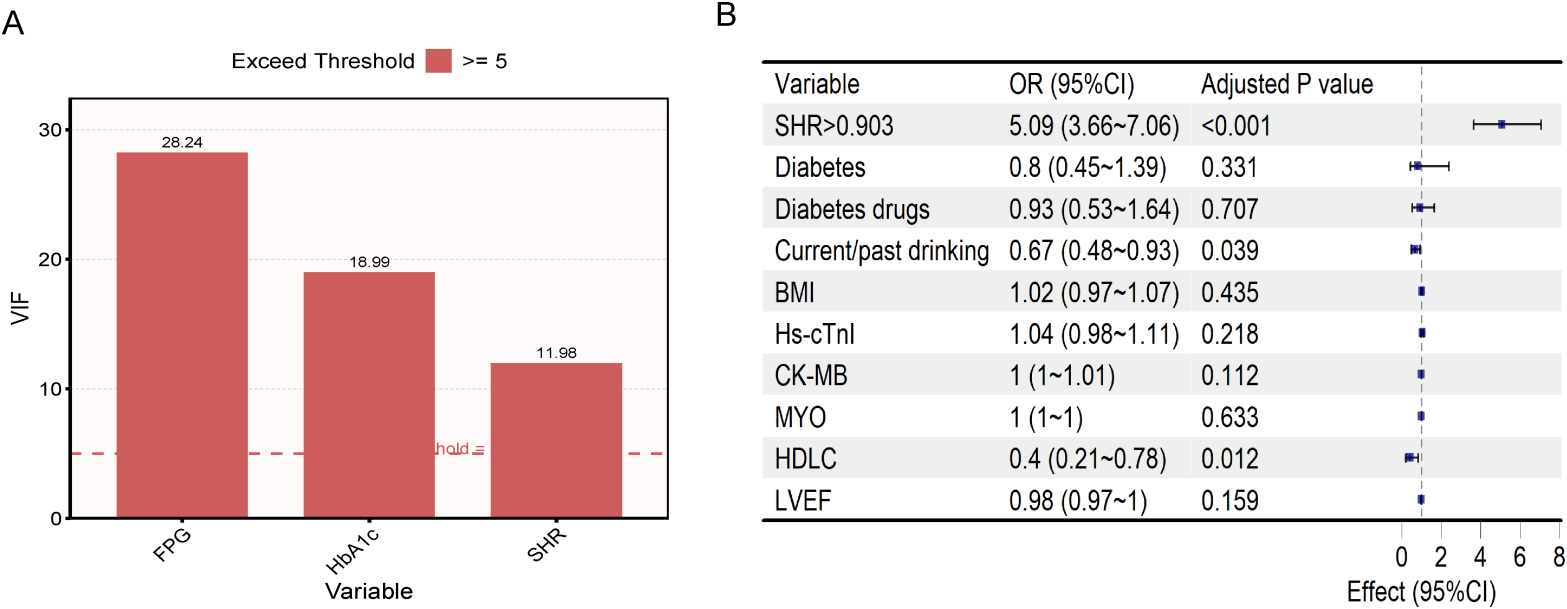
Collinearity assessment and multivariable model. **(A)** Variance inflation factors for FPG, HbA1c, and SHR. The dashed line indicates the prespecified threshold of 5. **(B)** Forest plot of adjusted associations with ATO. Squares indicate adjusted ORs and horizontal lines indicate 95% CIs; the table reports adjusted P values.

Covariates were selected based on clinical relevance, previous evidence, and their potential associations with both SHR and ATO. Because FPG, HbA1c and SHR exhibited substantial multicollinearity, with variance inflation factors of 28.24, 18.99 and 11.98, respectively (**Figure 4A**), FPG and HbA1c were not simultaneously included with SHR in the final model. After adjustment, SHR >0.903 remained independently associated with markedly higher odds of acute total occlusion (adjusted OR, 5.09; 95% CI, 3.66–7.06; P<0.001; **Table 3** and **Figure 4B**). Current or past drinking (adjusted OR, 0.67; 95% CI, 0.48–0.93; P = 0.039) and higher HDL-C levels (adjusted OR, 0.40; 95% CI, 0.21–0.78; P = 0.012) were independently associated with lower odds of acute total occlusion. By contrast, the associations of diabetes, diabetes drugs, BMI, hs-cTnI, CK-MB, myoglobin and LVEF observed in the univariable analysis were attenuated and were no longer statistically significant after multivariable adjustment (all P>0.05). The absence of an association between symptom-to-admission time and ATO may reflect the heterogeneous timing of thrombotic occlusion or the influence of collateral circulation.

**Table 3:** Multivariable logistic regression analysis of risk factors associated with ATO.

Variable	Adjusted OR (95% CI)	Adjusted P value
SHR>0.903	5.09 (3.66~7.06)	<0.001
Diabetes	0.8 (0.45~1.39)	0.331
Diabetes drugs	0.93 (0.53~1.64)	0.707
Current/past drinking	0.67 (0.48~0.93)	0.039
BMI	1.02 (0.97~1.07)	0.435
Hs-cTnI	1.04 (0.98~1.11)	0.218
CK-MB	1 (1~1.01)	0.112
MYO	1 (1~1)	0.633
HDL-C	0.4 (0.21~0.78)	0.012
LVEF	0.98 (0.97–1)	0.159

These findings suggest that the association of SHR for ATO is not solely attributable to the presence of diabetes mellitus and remains robust after accounting for potential collinearity between diabetes-related glycemic indicators.

### 3.3 Nonlinear association between SHR and acute total occlusion

**Figure 5:**
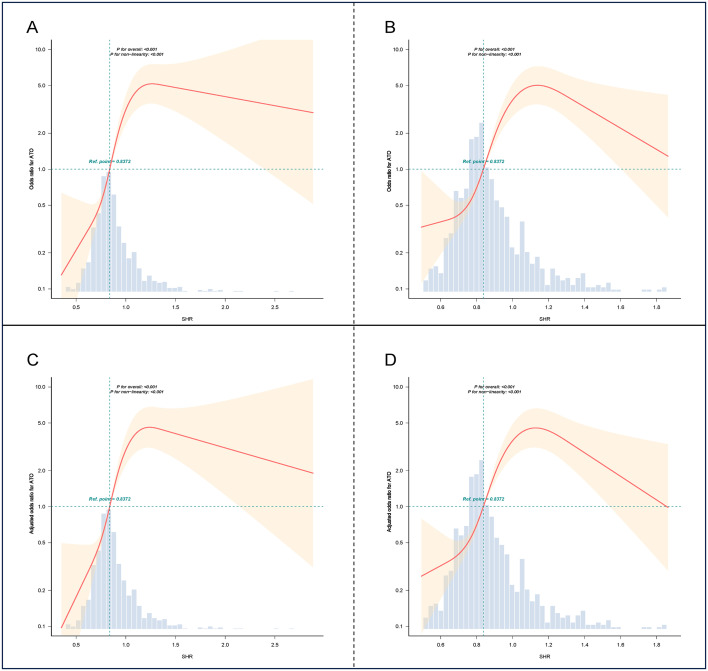
Nonlinear association between SHR and ATO. Solid lines show estimated ORs and shaded areas show 95% CIs. **(A)** Unadjusted model using the full distribution. **(B)** Unadjusted model restricted to the 1st–99th percentile range. **(C)** Model adjusted for T2DM, Diabetes drugs, BMI, age, sex, HDL-C, LVEF, Myocardial injury markers. **(D)** Adjusted model restricted to the 1st–99th percentile range. Histograms show the SHR distribution.

Restricted cubic spline analyses showed significant overall and nonlinear associations between SHR and the odds of ATO in the unadjusted model (both P<0.001; **Figure 5A**). To reduce the influence of extreme SHR values on curve estimation, the analysis was repeated after restricting SHR to the 1st–99th percentile range, and the nonlinear association remained evident (both P<0.001; **Figure 5B**). Similar results were observed after adjustment for T2DM, Diabetes drugs, BMI, age, sex, HDL-C, LVEF, Myocardial injury markers (both P<0.001; **Figure 5C**), as well as in the adjusted analysis restricted to the 1st–99th percentile range (both P<0.001; **Figure 5D**). Restricting SHR to the 1st–99th percentile range reduced the influence of these extreme values and supported the robustness of the overall nonlinear association.

## 4 Discussion

In this retrospective cohort of 827 patients with NSTEMI, elevated SHR at hospital admission was strongly and independently associated with ATO at coronary angiography. Patients with SHR >0.903 had approximately fivefold higher adjusted odds of ATO than those with SHR ≤0.903, and restricted cubic spline analysis demonstrated a robust nonlinear association across the SHR distribution.

Our findings build upon and extend the literature on stress hyperglycemia and coronary outcomes in acute coronary syndromes. Previous studies have consistently shown that stress hyperglycemia is associated with impaired myocardial perfusion and adverse outcomes after acute myocardial infarction (17, 18). However, absolute glucose values are influenced by chronic glycemic exposure, making it difficult to isolate the acute component of hyperglycemia in patients with pre-existing diabetes or impaired glucose metabolism—a condition present in 49.8% of our cohort. SHR addresses this limitation by normalizing acute glycemia to the chronic glycemic state estimated from HbA1c.

The superiority of SHR over HbA1c for discriminating ATO in our study is particularly informative. Although HbA1c was associated with ATO in univariable analysis, FPG and HbA1c were not entered together with SHR in the final model because they are mathematical components of SHR and exhibited substantial multicollinearity. The findings therefore support the association of relative, rather than chronic, hyperglycemia with ATO, but do not establish statistical superiority of SHR over HbA1c after simultaneous adjustment. This interpretation is consistent with reports of the prognostic value of SHR in coronary artery disease and in patients with STEMI undergoing PCI (11, 14).

The biological plausibility of SHR as a marker of ATO is supported by several mechanisms. Stress hyperglycemia is associated with prothrombotic and inflammatory responses, endothelial dysfunction, and impaired microvascular perfusion, all of which may accompany severe acute myocardial ischemia (19, 20). In addition, SHR may reflect the magnitude of adrenergic and counter-regulatory activation during acute illness (9). Because the present study was observational and SHR was measured at admission, however, these mechanisms remain hypotheses and reverse causation cannot be excluded.

Restricted cubic spline analysis further demonstrated a significant nonlinear association between SHR and the odds of ATO. Across both unadjusted and adjusted models, a trend toward higher odds was observed across the mid-range of SHR. This pattern remained evident after restricting the analysis to the 1st–99th percentile range, indicating that the association was not driven solely by extreme SHR values. At higher SHR levels, however, the shape of the fitted curve varied across model specifications and the confidence intervals widened substantially because of sparse data. Therefore, these findings should not be interpreted as establishing a precise biological threshold or a causal mechanism. Together, these complementary analyses support a robust, nonlinear association between elevated SHR and ATO. A nonlinear association between SHR and subsequent adverse outcomes has also been reported in a large AMI cohort (8), although prospective studies are still needed to validate the present cutoff and determine its clinical utility.

Because FPG and HbA1c are mathematical components of SHR, their simultaneous inclusion in the multivariable model introduced substantial multicollinearity. They were therefore excluded from the final SHR-based model. Consequently, the present analysis does not establish that absolute glucose levels provide risk information independent of SHR, nor does it demonstrate that combining FPG with SHR improves risk stratification. Nevertheless, SHR showed better discrimination for ATO than FPG in the overall cohort, suggesting that normalization of acute glycemia to chronic glycemic status may capture clinically relevant information beyond FPG alone. Whether SHR provides incremental association over conventional clinical variables and glycemic markers should be evaluated using externally validated models with formal assessments of calibration, reclassification, and clinical utility.

Higher HDL-C remained independently associated with lower odds of ATO after multivariable adjustment. This finding is consistent with the proposed anti-inflammatory, endothelial-protective, and antithrombotic properties of HDL particles (21). However, the observational design precludes a causal or mechanistic interpretation, and HDL-C may also reflect broader metabolic or cardiovascular risk profiles not fully captured by the measured covariates. LVEF was lower in patients with SHR >0.903 than in those with SHR ≤0.903, but this association was attenuated after multivariable adjustment. The lower LVEF observed in the high-SHR group may therefore reflect greater overall disease severity or the higher prevalence of ATO rather than an independent effect of stress hyperglycemia on myocardial function. However, as an observational finding, this association may be confounded by unmeasured lifestyle or therapeutic factors and should not be interpreted as causal.

Although SHR is readily available, the present results do not support its use as a stand-alone criterion for urgent coronary angiography, revascularization, or intensive glucose-lowering therapy. SHR should be considered a complementary marker, and its incremental value beyond established clinical and electrocardiographic assessment requires external validation.

Several limitations of this study should be acknowledged. First, the retrospective, single-center design may have introduced selection bias and may limit the generalizability of the findings to other populations and clinical settings. Second, ATO was primarily identified by visual assessment of coronary angiography, which is inherently subject to interobserver variability. Patients with CTO were deliberately excluded from the present analysis to maintain a clear focus on acute thrombotic occlusion. Although excluding definite CTO reduced contamination of the ATO group by clearly chronic occlusions, this approach may have introduced selection bias and limited the applicability of our findings to patients with uncertain occlusion timing or angiographic features suggestive of chronicity. Furthermore, distinguishing ATO from CTO by angiography alone can be challenging, and residual misclassification between acute and chronic coronary occlusion cannot be completely excluded, despite independent review by experienced interventional cardiologists. Third, admission FPG levels may have been influenced by acute physiological stress, and residual confounding from unmeasured clinical factors cannot be excluded. The interval between admission blood sampling and coronary angiography may have resulted in changes in culprit-vessel patency, potentially causing misclassification of ATO status relative to the time of SHR measurement. Finally, the lack of external validation and long-term follow-up precluded assessment of the reproducibility of the identified SHR cutoff and its association with long-term clinical outcomes.

## 5 Conclusions

In this retrospective cohort, elevated admission SHR was independently and nonlinearly associated with ATO. Although SHR may complement clinical assessment, further external validation is required before clinical implementation.

## Data Availability

The original contributions presented in the study are included in the article/supplementary material. Further inquiries can be directed to the corresponding author.
